# 2-Methoxyestradiol Alleviates Experimental Autoimmune Uveitis by Inhibiting Lymphocytes Proliferation and T Cell Differentiation

**DOI:** 10.1155/2016/7948345

**Published:** 2016-05-08

**Authors:** Linxinyu Xu, Tianshu Yang, Shaobo Su, Fang Wang

**Affiliations:** ^1^Department of Ophthalmology, Shanghai Tenth People's Hospital, Tongji University School of Medicine, Shanghai 200092, China; ^2^Shanghai Tenth People's Hospital, Tongji University School of Medicine, 301 Middle Yanchang Road, Shanghai 200092, China

## Abstract

*Purpose*. To investigate the effect of 2-Methoxyestradiol (2ME2) on experimental autoimmune uveitis (EAU) and the mechanism.* Method*. C57BL/6 male mice were used to establish the EAU model. 2ME2 was abdominal administrated in D0–D13, D0–D6, and D7–D13 and control group was given vehicle from D0–D13. At D14, pathological severity was scored. Lymphocyte reaction was measured using MTT assay. T cell differentiation in draining lymph nodes and eye-infiltrating cells was tested by flow cytometry. Proinflammatory cytokines production from lymphocytes was determined by ELISA.* Result*. The disease scores from 2ME2 D0–D13, 2ME2 D0–D6, 2ME2 D7–D13, and vehicle groups were 0.20 ± 0.12, 1.42 ± 0.24, 2.25 ± 0.32, and 2.42 ± 0.24. Cells from all 2ME2 treated groups responded weaker than control (*p* < 0.05). The inhibitory effect of 2ME2 on lymphocyte proliferation attenuated from 2ME2 D0–D13 to 2ME2 D0–D6 and to 2ME2 D7–D13 groups (*p* < 0.05). 2ME2 treated mice developed fewer Th1 and Th17 cells both in draining lymph nodes and in eyes than control (*p* < 0.05). Lymphocytes from 2ME2 group secreted less IFN-*γ* and IL-17A than those from control (*p* < 0.05).* Conclusion*. 2ME2 ameliorated EAU progression and presented a better effect at priming phase. The possible mechanism could be the inhibitory impact on IRBP specific lymphocyte proliferation and Th1 and Th17 cell differentiation.

## 1. Introduction

Uveitis is a group of diseases among the leading causes of visual deficit and blindness. Uveal and retinal layers as well as vitreous cavity could all be affected within disease progression. Clinically, this group of diseases can be divided into infectious and noninfectious causes, the latter one is known as autoimmune uveitis [1]. Due to the limitation of obtaining biopsy samples from active uveitis patients, the underlying pathological progress of different types of uveitis still remains unknown. However, immune responses led by abnormal T cell which recognize uveal antigens are the main process during disease development [2]. Nowadays, uveal and retinal antigens, especially interphotoreceptor retinoid binding protein (IRBP), were widely used in uveitis animal models induction [3]. Among them, experimental autoimmune uveitis (EAU) is one of the most stable and regularly used disease models.

Different immune cells subsets play important roles in disease development of EAU. Th1 and Th17 responses and relative cytokines are believed to be pathogenic in tissue damage [4, 5]. Th1 cells, as is known, by secreting interferon-gamma (IFN-*γ*) can activate macrophages, while Th17 cells by secreting interleukin 17A (IL-17A) promote inflammatory responses [6, 7]. Many efforts had been done to inhibit either the T cell differentiation towards pathogenic cells or the subsequent inflammatory cytokine act in the inner ocular milieu [8].

Endogenous metabolite of estrogen, 2-Methoxyestradiol (2ME2), is a promising antiangiogenic and antitumor compound [9–12]. Besides its disturbance on tubulin formation, the anti-inflammatory effect of it was also proved in damping macrophage function and antigen presenting ability of NK cells [13]. In experimental autoimmune encephalomyelitis (EAE), 2ME2 exhibited disease modifying effect via inhibiting T cell activation [14]. Moreover, pregnancy was found to be a protective factor in rodent EAU model induction [15], while investigation in periparturition women revealed that pregnancy can lower the risk of developing uveitis [15]. Other than estradiol, 2ME2 levels in plasma and urine are highly elevated during late pregnancy [16, 17]; could it affect autoimmune disease development, especially EAU development, remains uncertain.

In this study, we demonstrated that the endogenous methylated estrogen, 2ME2, could taper autoimmune uveitis development in a time-dependent manner. The disease modifying effect was established on reducing antigen specific T cell proliferation, inhibiting T cell differentiation and related proinflammatory cytokines secretion.

## 2. Methods

### 2.1. Mice

6~8-week-old C57BL/6 mice were purchased from Shanghai Laboratory Animal Center, CAS (SLACCAS), and housed in a specific-pathogen-free condition with water and standard laboratory chow ad libitum. Animal care and use were in compliance with the Institutional Animal Care and Use Committee Guidelines and the Association for Research in Vision and Ophthalmology Statement for the Use of Animals in Ophthalmic and Vision Research.

### 2.2. Material and Reagents

Three peptides of IRBP protein were employed to formulate the antigen complex. They were 1–20 (SGIPYIISYLHPGNTILHVD), 461–480 (LRHNPGGPSSAVPLLLSYFQ), and 651–670 (LAQGAYRTAVDLESLASQL) which C57BL/6 mice were sensitive to [3]. Peptides were purchased from Sangon Biotech and emulsified 1 : 1 vol/vol in CFA (Sigma) containing 2.5 mg/mL* Mycobacterium tuberculosis* (Difco). Three peptides were used: 1–20 300 *μ*g, 461–480 200 *μ*g, and 651–670 200 *μ*g per mouse. 2ME2 purchased from MedChem Express was dissolved into DMSO (Sigma). Roswell Park Memorial Institute 1640 (RPMI1640) medium was purchased from Gibco. MTT kit was purchased from Sigma. ELISA kit was purchased from eBioscience. CD3, CD4, IL-17A, and IFN-*γ* antibodies used in flow cytometry were purchased from BD. Other reagents and materials used in cell culture, flow cytometry, and HE stain were purchased from Sigma unless otherwise specified.

### 2.3. Induction of EAU and Group Design

C57BL/6 mice were immunized subcutaneously 0.1 mL at tail and 0.05 mL at both thigh sites with IRBP antigen complex. 500 ng Pertussis toxin was injected concurrently. This day was settled as day 0. Then mice were divided into 4 groups, each group containing 5 mice. 15 mg/kg 2ME2 or vehicle was abdominal injected during 0–13 days, 0–6 days, and 7–13 days. At day 14 eyes or lymphoglandula was collected after euthanasia.

### 2.4. Histopathology and Scoring of EAU

Eyes harvested 14 days after immunization were prefixed in 4% phosphate-buffered glutaraldehyde for 1 h (to prevent artifactual detachment of the retina) and then transferred to 10% phosphate-buffered formaldehyde until processing. Fixed and dehydrated tissue was embedded in methacrylate, and 4 to 6 *μ*m sections were stained with standard H&E. Eye sections cut through pupillary-optic nerve planes were scored in a masked fashion. Severity of EAU was graded on a scale of 0–4 in half-point increments using the criteria described previously [18], based on the type, number, and size of lesions.

### 2.5. Flow Cytometry

Eyes' external tissue was trimmed after enucleation; incision was made along the limbus to remove the lens. The rest of the eye was minced into small pieces and digested in 1 mg/mL collagenase IV in RPMI containing 10% FCS for 1 hour at 37°C. After trituration and filtration, the resultant cell pellet was resuspended in RPMI with 10% FCS and seeded in 48-well plate. Harvested lymph nodes were dispersed by trituration in RPMI with 10% FCS on ice, followed by filtration and washing. Then single-cell suspension was obtained and seeded in 96-well plate for intracellular cytokine evaluation and proliferation assay. PMA (50 ng/mL) and ionomycin (500 ng/mL) together with brefeldin A were added to pulse both the eye-infiltrating cells and lymphocytes for 5 hours. Cells were then fixed with 2% paraformaldehyde, permeabilized in 0.5% saponin, and stained with CD4, IFN-*γ*, and IL-17A [19]. Analysis was performed on FACS Verse*™* (BD).

### 2.6. MTT Assay

MTT assay was used to determine the effect of IRBP on cell proliferation. Cells from 4 groups previously described were seeded 1.5*∗*10^6^/well and maintained with 200 *μ*L culture media and 5 *μ*g/mL IRBP for 48 hours in 96-well plates. 80 *μ*L MTT was then added to each well and cells were further incubated for 3 hours at 37°C. Supernatants were then removed and 100 *μ*L of chromogenic reagent was added to each well. After shaking in darkness for 10 min, a spectrophotometer was used to measure absorbance at 540 nm. Three independent experiments were performed.

### 2.7. ELISA

Supernatants of lymphocytes pulsed by IRBP antigen complex for 48 hours were harvested and tested for IL-17A and IFN-*γ* concentration according to the manuals from the manufacturer. Briefly, ELISA plate was coated with 100 *μ*L/well of capture antibody in 1x coating buffer, sealed, and incubated overnight at 4°C. After 3 times wash, plate was then blocked using ELISA/ELISPOT diluent at room temperature for 1 hour. Standard proteins as well as supernatant samples were prepared and 100 *μ*L/well was added to the plate. Each sample was triplicated. Then the plate was sealed and incubated at room temperature for 2 hours. All wells were washed 5 times afterwards. 100 *μ*L/well IL-17A or IFN-*γ* antibody was then added and incubated at room temperature for 1 hour. After 5 times aspiration, 100 *μ*L/well of diluted Avidin-HRP was added and incubated at room temperature for 30 minutes. Wells were then soaked in wash buffer for 2 minutes prior to aspiration. 100 *μ*L/well of TMB solution was added to each well after aspiration. Plate was incubated for 15 minutes and 50 *μ*L of stop solution was added to each well. Finally plate was read at 450 nm, and concentration of samples was calculated according to the standard curve.

### 2.8. Data Analysis

All experiments were repeated at least three times. Data were expressed as mean ± SEM. The statistical analysis was carried out using Student's *t*-test. A *p* value of 0.05 or less was considered statistically significant.

## 3. Results

### 3.1. 2ME2 Ameliorated EAU Progression

**Figure 1:**
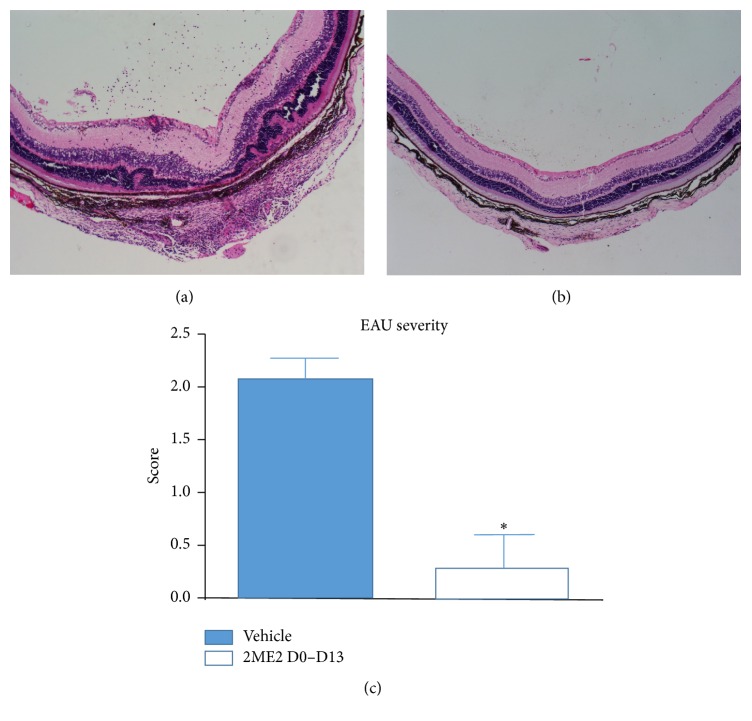
Effects of 2ME2 on EAU mice. (a) Retina HE section from vehicle treated EAU mice, scored 3. (b) Retina HE section from 2ME2 treated EAU mice, scored 0. (c) Disease score from 2ME2 D0–D13 group was lower than that from vehicle treated group (*∗* indicated a *p* < 0.05), each group containing 5 mice.

To investigate the effect of 2ME2 on uveitis development, C57BL/6 mice were randomly assigned into two groups and immunized with IRBP peptide. 2ME2 group started 2ME2 (15 mg/kg) intraperitoneally from day 0 to day 13 while control group was given with vehicle. On day 14, when inflammation was believed to reach the peak, eyes were harvested for blind histopathological examination. Eyes from control group presented with severe structure destruction, abundant inflammatory cells infiltration both in vitreous body and in retina, vasculitis, photoreceptor layer damage, and moderate to large retinal folds were noticed in almost every case (Figure 1(a)), while 2ME2 treated eyes maintained a normal structure and few cells infiltrated into retina (Figure 1(b)). The disease score of 2ME2 group was 0.30 ± 0.30, significantly lower than that of control group 2.09 ± 0.28 (*p* < 0.05), each group containing 5 mice (Figure 1(c)).

### 3.2. Time-Dependent Effect of 2ME2 on EAU Development

**Figure 2:**
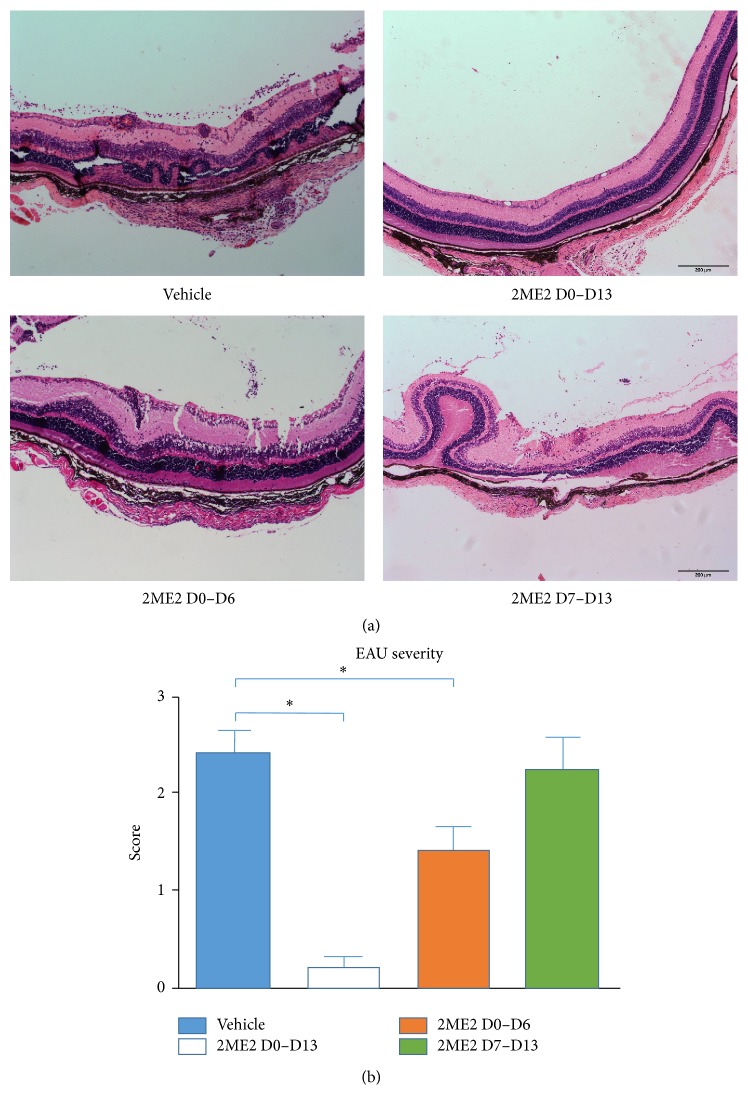
Time-dependent 2ME2 effects on EAU mice. (a) Retina HE sections from 2ME2 and vehicle treated groups. Vehicle, scored 3. 2ME2 D0–D13, scored 0. 2ME2 D0–D6, scored 1.5. 2ME2 D7–D13, scored 2.5. (b) Disease score from 2ME2 D0–D13 group was the lowest among the four groups, followed by 2ME2 D0–D6 group and 2ME2 D7–D13 group and vehicle group scored the highest (*∗* indicated a *p* < 0.05), each group containing 5 mice.

EAU mice immunized using active method undergo priming phase (day 0 to day 6) and effector phase (day 7 to day 13) to develop uveitis. To examine 2ME2 effect on uveitis development in different phases, 2ME2 (15 mg/kg) was intraperitoneally administrated either from day 0 to day 6, from day 7 to day 13, from day 0 to day 13, or vehicle. Compared to control group, HE sections from 2ME2 D0–D13 group remained almost normal retinal structure and exhibited the best curative outcome; those from 2ME2 D0–D6 group had less cell infiltration, milder vasculitis, and less structure disruption, while those from 2ME2 D7–D13 exhibited no much remission in observation (Figure 2(a)). The disease scores from 2ME2 D0–D13, 2ME2 D0–D6, 2ME2 D7–D13, and vehicle groups were 0.20 ± 0.12, 1.42 ± 0.24, 2.25 ± 0.32, and 2.42 ± 0.24, each group containing 5 mice. The differences between 2ME2 D0–D13 and vehicle groups and 2ME2 D0–D6 and vehicle groups were both significant (*p* < 0.05).

### 3.3. 2ME2 Attenuates Ag-Specific Immune Responses

**Figure 3:**
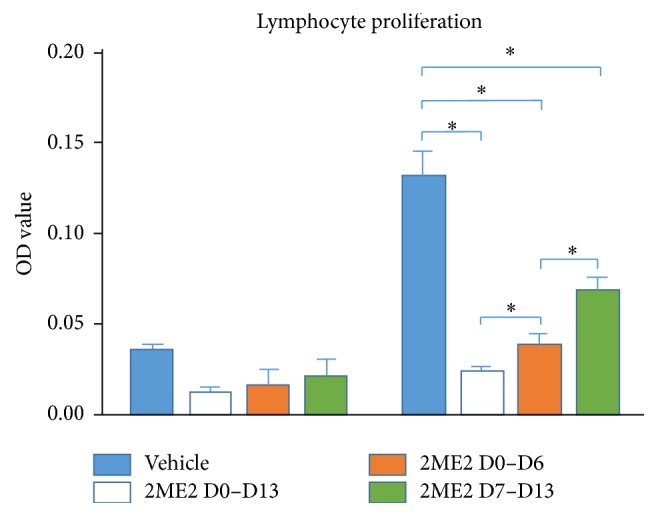
Lymphocytes harvested from 2ME2 treated EAU mice and vehicle mice, seeded 1.5*∗*10^6^ cells per well in 96-well plate (triplicated each sample) and incubated within 5 *μ*g/mL Ag concentration for 48 h. Cell number was evaluated using MTT assay. Lymphocytes from vehicle group proliferated much faster than 2ME2 treated groups. Besides the weakest proliferation rate in 2ME2 D0–D13 group, 2ME2 D0–D6 group cells number was lower than that of 2ME2 D7–D13 group (*∗* indicated a *p* < 0.05).

To assess the inhibitory effect of 2ME2 on Ag-specific immune response, lymphocyte proliferation method was used. Draining lymph node cells from 2ME2 D0–D13, 2ME2 D0–D6, 2ME2 D7–D13, and vehicle groups were pulsed with IRBP for 48 h and cell proliferation was measured using MTT assay. Cells from all 2ME2 treated groups responded significantly weaker than control (*p* < 0.05). The inhibitory effect of 2ME2 on lymphocyte proliferation attenuated from 2ME2 D0–D13 to 2ME2 D0–D6 and to 2ME2 D7–D13 groups (*p* < 0.05) (Figure 3).

### 3.4. 2ME2 Inhibits Th1 and Th17 Differentiation

**Figure 4:**
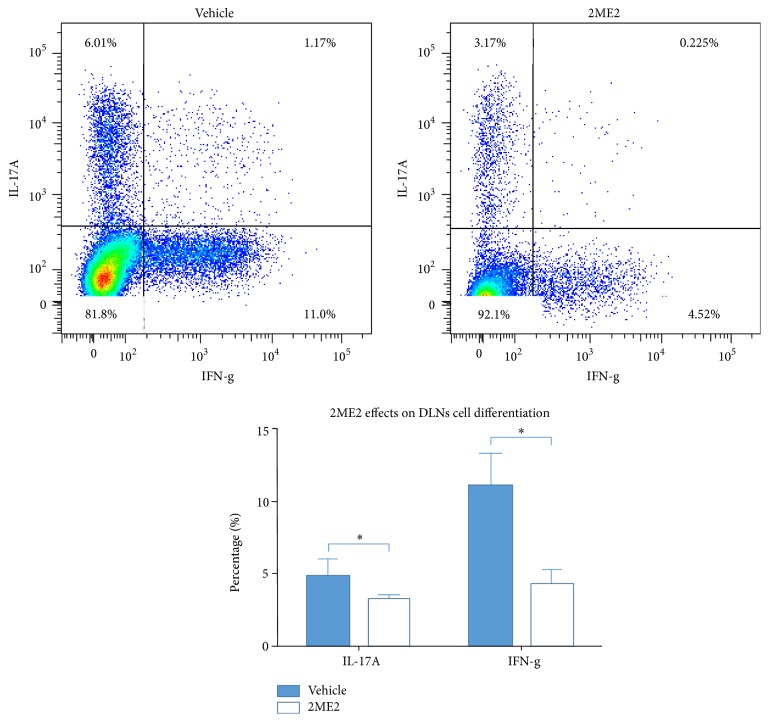
Effects of 2ME2 on draining lymph nodes cells differentiation. Flow cytometry figure was from one mouse. Both Th1 (IFN-g) and Th17 (IL-17A) cells percentages were lower in 2ME2 group than those in vehicle group (*∗* indicated a *p* < 0.05), each group containing 5 mice.

**Figure 5:**
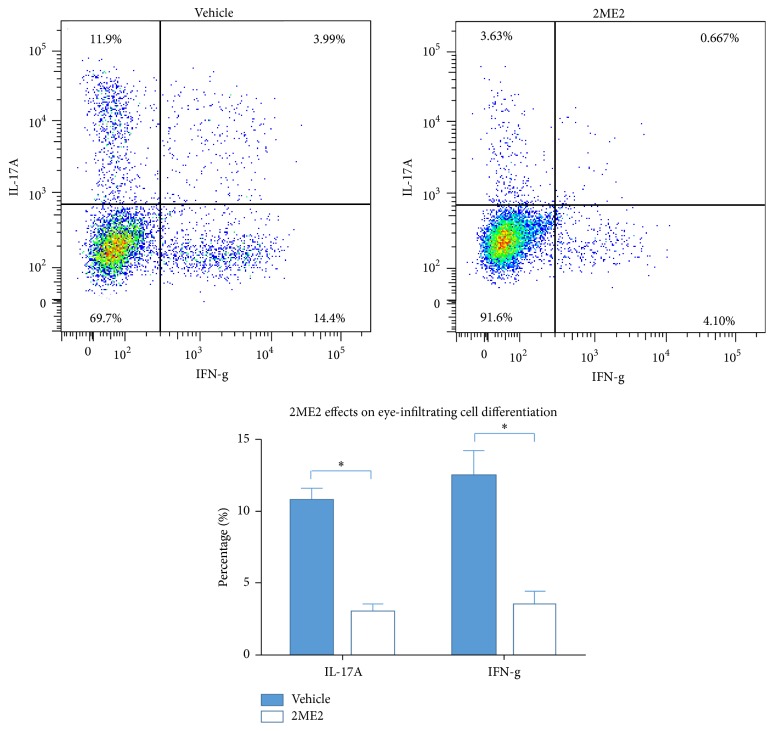
Effects of 2ME2 on eye-infiltrating cells differentiation. Flow cytometry figure was from one mouse. Both Th1 (IFN-g) and Th17 (IL-17A) cells percentages were lower in 2ME2 group than those in vehicle group (*∗* indicated a *p* < 0.05), each group containing 5 mice.

**Figure 6:**
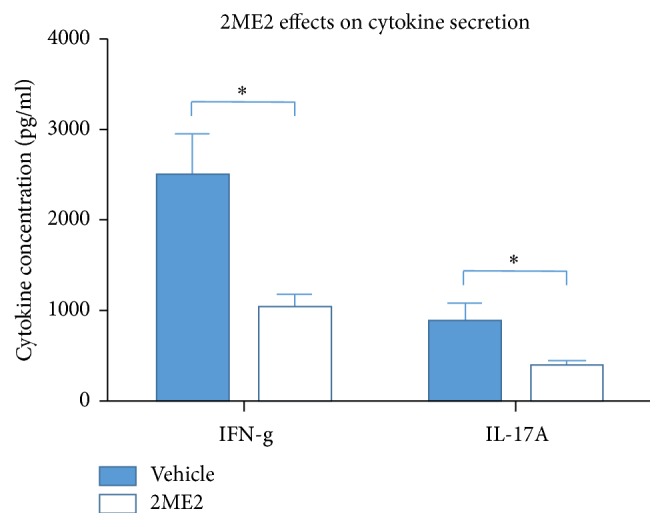
Effects of 2ME2 on cytokines secretion. Both IFN-g and IL-17A levels were lower in 2ME2 group than those in vehicle group (*∗* indicated a *p* < 0.05).

To further investigate the disease modifying mechanism of 2ME2, draining lymph nodes cells and eye-infiltrating cells were collected and analyzed using flow cytometry described above. 2ME2 treated mice developed fewer Th1 cells 4.22 ± 1.06% and Th17 cells 3.21 ± 0.32% in draining lymph nodes than 11.10 ± 2.26% Th1 cells and 4.80 ± 1.21% Th17 cells in vehicle group (*p* < 0.05), each group containing 5 mice (Figure 4). Similar results were obtained in eye-infiltrating cells analysis. 2ME2 treated mice developed 3.47 ± 0.91% Th1 cells and 2.93 ± 0.54% Th17 cells in eyes, while vehicle group developed 12.42 ± 1.80% Th1 cells and 10.80 ± 0.81% Th17 cells (*p* < 0.05), each group containing 5 mice (Figure 5). In line with these results, lymphocytes from 2ME2 group secreted more IFN-*γ* and IL-17A than those from control after 48 h antigen induction (*p* < 0.05) (Figure 6). Together, 2ME2 could damper T cells function via inhibiting Th1 and Th17 differentiation.

## 4. Discussion

The pathological mechanism during clinical uveitis progression is much more sophisticated than what we have already learnt from EAU models. However, from pathological view, they share lots of similarities including subretinal and choroidal granuloma, retinal vasculitis, retinal detachment, and infiltrating cells both in vitreous cavity and in retina [20]. In this study, anti-inflammatory effect of 2ME2 was examined using EAU models established in male C57BL/6 mice. 2ME2 exhibited different efficacy during different phases of the disease. Further, lymphocyte proliferation as well as T cell differentiation towards Th1 and Th17 lineages was determined, and the inhibitory effect of 2ME2 was discovered on both.

The antiproliferative and antimitogenic properties of 2ME2 were widely studied in carcinogenesis and angiogenesis [11, 21]. The reported disease modifying activity in autoimmune disorders of this endogenous metabolite of estradiol gave us the hint that 2ME2 might regulate EAU progression as well [22]. 2ME2 presented protective effect in collagen-induced arthritis (CIA) model and EAE models administrated either intraperitoneally or orally [14]. IRBP/CFA induced EAU model shares many key disease drivers with CIA and EAE. All of them were mediated by Th1 and Th17 cell development and cytokine production [23]. After 14 days of 2ME2 administration, severity of uveitis in EAU mice was greatly reduced in observation comparing to vehicle group.

EAU underwent priming phase and effector phase to reach the peak of inflammation. To discover whether the efficacy of 2ME2 was time-dependent, 2ME2 was injected on D0–D6, D7–D13, and D0–D13 separately. Besides the best curative effect in 2ME2 D0–D13 group, priming phase drug employment exerted better outcome than those administrated in effector phase. From these results, we could see that it was more important for 2ME2 to perform its inhibitory effect on priming phase. In priming phase, uveitogenic antigen was processed and presented to T cell, resulting in T cell proliferation. The underlying mechanism of superior efficacy of 2ME2 in priming phase might be related to these processes.

In observation of antigen specific lymphocyte proliferation, 2ME2 can significantly inhibit response of IRBP specific lymphocyte harvested from EAU mice. This effect could be maximized in 2ME2 D0–D13 group followed by 2ME2 D0–D6 group, whereas 2ME2 D7–D13 group has the moderate response, only weakened compared to control group. This result is in line with histopathological findings and together proved that a more important effect of 2ME2 is exerted in priming phase. In other researches, 2ME2 can reduce macrophage and NK cells activity, inhibiting T cell activation and proliferation [24, 25]. Having the above results, the hypothesis, whether 2ME2 could regulate T cell differentiation, was then elicited.

By isolating lymphocytes from draining lymph nodes and eye-infiltrating cells, T cell differentiation towards Th1 and Th17 lineages was determined to be inhibited by 2ME2 via flow cytometry. Proinflammatory cytokines, IFN-*γ* and IL-17A, were also downregulated in this process, reducing effector phase T cell mediated target tissue destruction. When activated using costimuli, 2ME2 incubated T cells expressed lower levels of IFN-*γ* and IL-17A [14]. This is in agreement with our in vivo results.

Previous studies proved that either Th1 or Th17 cells can independently drive specific effector responses during autoimmune diseases development [4]. Targeting either Th1 or Th17 cells may shift the balance of lineage specific response towards the alternative one without minimizing pathological changes. Comparing to targeting only one T cell lineage response, our study revealed that 2ME2 can equally inhibit both Th1 and Th17 responses, implying a bright future of 2ME2 for clinical application.

## 5. Conclusion

2ME2 ameliorated EAU progression and presented a better effect at priming phase. The possible mechanism of this effect could be the inhibitory impact on IRBP specific lymphocyte proliferation and Th1 and Th17 cell differentiation.
